# What is the balance of benefits and harms for lung cancer screening with low-dose computed tomography?

**DOI:** 10.1177/0141076821991108

**Published:** 2021-03-10

**Authors:** Stephen H Bradley, Bethany Shinkins, Martyn PT Kennedy

**Affiliations:** 1 Leeds Institute of Health Sciences, 4468 University of Leeds, Leeds LS2 9JT, UK; 2 Test Evaluation Group, Academic Unit of Health Economics, 4468 University of Leeds, Leeds LS2 9JT, UK; 3 Department of Respiratory Medicine, 4472 Leeds Teaching Hospitals NHS Trust, Leeds LS9 7TF, UK

After the National Lung Screening Trial (NLST)^1^ demonstrated improved lung cancer mortality almost a decade ago, hopes have been raised that low-dose computed tomography could be used to detect lung cancer in asymptomatic populations and improve outcomes by reducing the number of people diagnosed with advanced disease. The publication of the long-awaited Nederlands-Leuvens Longkanker Screenings Onderzoek (NELSON) trial last year^2^ has provided further evidence of a lung cancer mortality benefit and has provoked further calls to implement national population-based screening programmes, but the case for lung cancer screening remains controversial.

## Evidence for the benefits of screening

The National Lung Screening Trial randomised over 53,000 participants, aged between 55 and 74 years with at least a 30 pack-year smoking history, to three annual screening rounds with either chest X-ray or low-dose computed tomography.^1^ At a median follow-up of 6.5 years, a relative reduction in lung cancer mortality of 20% in the low-dose computed tomography arm (247/100,000 person-years) compared to the chest X-ray arm (309/100,000 person-years) was found. A total of 320 screens were required to prevent one lung cancer death (falling to 303 at 12.3 years),^3^ a figure comparable to estimates for breast cancer screening.^4,5^ The National Lung Screening Trial is also the only screening trial to have demonstrated an all-cause mortality benefit (6.7%, 95% CI 1.2–13.6); however, this finding was no longer statistically significant in the recently published extended follow-up (median 12.3 years follow-up).^3^

Several smaller trials conducted in Europe were not powered to determine any mortality benefit^6–11^ and heterogeneity in study design has hindered the prospect of pooling these results. With almost 16,000 participants followed up for 10 years, the NELSON trial was published in February 2020 and is the only trial of low-dose computed tomography screening aside from the National Lung Screening Trial which was adequately powered to demonstrate a lung cancer mortality benefit.^2^ Participants were randomised to either low-dose computed tomography or routine care. Findings were broadly comparable to the National Lung Screening Trial, despite the differing comparators (the National Lung Screening Trial used a chest X-ray as a control), with a relative reduction in lung cancer mortality of 24% at 10 years of follow-up (95% CI: 6–39) for men.^12^ Moreover, 92–133 participants needed to be screened per round to prevent one lung cancer death.^13^

NELSON was not powered to detect an improvement in all-cause mortality. Arguably this is an unrealistic benchmark since it has been estimated that it would require 80,000 patients to be randomised and followed up for over a decade to demonstrate all-cause mortality benefits based on improved lung cancer outcomes alone.^14^ The NLST and NELSON studies are summarised in table 1.

## Evidence for the harms of screening

Nodules are found in around half of those screened for lung cancer. The smallest nodules are not associated with an increased risk of lung cancer compared to those with no nodules so do not require further assessment.^16^ The majority of those that require surveillance or investigation are found to be benign.^17^ In the National Lung Screening Trial, all but the smallest nodules were considered positive, resulting in false-positive results for 23.3% of all low-dose computed tomography scans performed. Reflecting modern surveillance protocols,^18,19^ NELSON added an ‘intermediate’ group for low-risk nodules. Almost 10% of all screens fell into this category and required a further follow-up screen in 3–4 months. Consequently, false positives in NELSON were nominally much lower overall at 1.2%, although over half of the positive results were still false positives (56.5%).^17^

In the National Lung Screening Trial, 0.06% of the false-positive tests in the low-dose computed tomography group were associated with a ‘major complication after an invasive procedure’.^1^ Complications which may have resulted from screening are yet to be reported for the NELSON trial.

The National Lung Screening Trial reported that ‘clinically significant’ abnormalities other than lung cancer were identified in 7.5% of the low-dose computed tomography scans. Incidental findings have been examined for 1929 participants of NELSON, 129 (6.8%) of whom had findings which required further evaluation or could have ‘substantial clinical implications’. Since only 21 (1.1%) were found to represent significant findings following further evaluation, the authors argue that searching for and reporting incidental findings should not be undertaken in low-dose computed tomography screening trials.^20^

A proportion of screen-detected cancers may never have presented symptomatically (over-diagnosis). At 6 years of follow-up, the National Lung Screening Trial report 18.5% of cases identified by low-dose computed tomography were due to over-diagnosis; however, this figure falls to 3.1% at 12 years. Over-diagnosis of broncho-alveolar cell carcinomas remained high at 79%.^3^ These lesions are identifiable as pure ground glass nodules on low-dose computed tomography and practice has evolved since the National Lung Screening Trial so that such cases are now followed up with surveillance^21^. 8.9% of screen-detected cancers in NELSON were attributed to over-diagnosis at 11 years, which the authors argue is likely to diminish if follow-up was extended further.

Some evidence exists that participation in lung cancer screening has negative psychological consequences^22,23^ and false-positive results seem likely to have some adverse effects on quality of life,^24^ at least in the short term. However, such effects seem to attenuate over time, resulting in no clinically significant long-term psychosocial harms.^25^

Radiation exposure over the three screening rounds in the National Lung Screening Trial has been estimated at 8 mSv. It is thought that screening could result in one death due to radiation per 2500 people screened over a 10- to 20-year period^26^ and that one radiation induced major cancer may be expected from every 108 lung cancers detected through screening.^27^

## Biases and uncertainties

Due to the many biases that can affect screening studies, some have argued that to be confident a screening intervention is beneficial, it should demonstrate a reduction in overall (all-cause) deaths rather than only a reduced number of lung cancer deaths. It has been argued that without showing an all-cause mortality reduction considering disease-specific mortality reductions alone may mask harms that result from screening.^28,29^ Although the National Lung Screening Trial did show a reduction in all-cause mortality, it has also been questioned whether a genuine benefit exists since lung cancer cases and cardiovascular disease identified by NLST cannot account for the overall reduction in mortality.^29,30^ As lung cancer causes a minority of deaths, even among smokers who meet eligibility criteria for the National Lung Screening Trial, a relative reduction in lung cancer mortality of 20% would only equate to an all-cause mortality reduction of at most 0.8%.^14^

Participants in studies, and indeed, screening programmes are likely to represent healthier cohorts within the populations at risk of developing cancer, with important consequences for outcomes.^31^ A comparison of National Lung Screening Trial participants, compared to those who would be eligible for lung cancer screening in the US, suggests that a ‘healthy volunteer’ effect was evident^32^ while NELSON participants have been considered broadly representative of the wider population.^33^

It is impossible to know for certain whether the reduced lung cancer deaths demonstrated in the National Lung Screening Trial and NELSON would correspond to real-world improvements in overall mortality. Both studies could have provided more detailed information on complications resulting from screening to help make that assessment. If the reported low rates of complications are taken at face value, it seems likely that the reduction in lung cancer mortality with screening would be greater than iatrogenic mortality.

## Only eligible participants with screen detected cancers can benefit

Lung cancer screening relies on the selection of individuals with sufficient risk to justify screening. A significant proportion of lung cancer cases, perhaps greater than 10%, occur in people who have no smoking history at all,^34^ and less than one-third of patients with lung cancer in the United States would have been eligible for screening under the National Lung Screening Trial protocol.^35^ Meanwhile, uptake for those who are eligible to participate in screening trials and pilots has been modest, at around 30%–60%.^2,11,36,37^ Therefore, only a minority of patients who develop lung cancer could be detected through screening.

For those who do participate and develop lung cancer, a minority will occur between screening rounds. Such ‘interval cancers’, which accounted for 12.8% of cases in NELSON, tend to be faster growing and more aggressive, with poorer outcomes than screen-detected cancers.^31^

## Informed decision making

**Figure 1.**
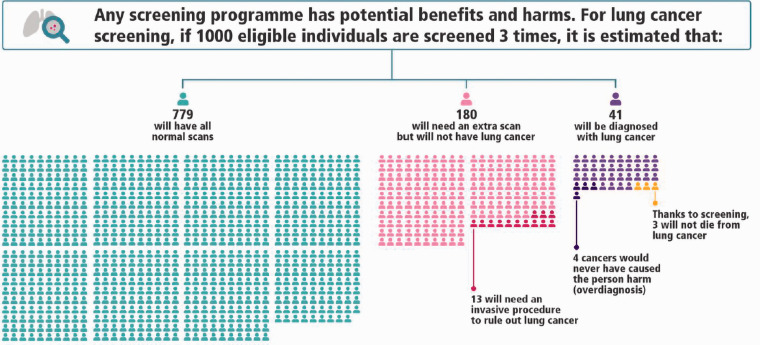
1000-person tool for lung cancer screening. The tool illustrates estimated outcomes based on in the National Lung Screening Trial (NLST). Since the NLST most nodules identified on low-dose computed tomography are followed up using a surveillance protocol, rather than immediate investigation. To reflect contemporary standards of care, this tool assumes the Lung-RADS protocol is used to interpret low-dose computed tomography results. A 1000-person tool has not yet been created to reflect the findings of the NELSON study. Reproduced with permission from the International Agency for Research on Cancer. Full page infographic available at: https://www.iarc.fr/infographics/benefits-and-harms-of-lung-cancer-screening/

It is now acknowledged that information provided to potential participants of screening should aim to convey potential benefits and harms, rather than persuade individuals to take part.^38–40^ Evidence from the US suggests that information provided by clinicians about screening is not currently adequate to support informed decision making.^41^ Adequate communication should be embedded into any proposed lung cancer screening programme, making use of existing decision aids (see Figure 1)^42,43^ and developing high-quality resources to support patient decision making.^44^

## What is current policy on lung cancer screening?

Lung cancer screening has been recommended by the United States Preventative Services Task Force since 2013^45,46^ and has been reimbursed through Medicare and Medicaid since 2015.^47,48^ However, only about 2% of eligible patients undergo screening.^49^ This could be due to the lack of central co-ordination of screening programmes, but may also reflect limited interest in screening from eligible individuals.

In Europe, some have called to prepare for lung cancer screening, even before the results of NELSON were published,^50–54^ while others have cautioned against anticipating the outcome of formal decision-making processes.^55^ Lung cancer screening has become particularly controversial in England, where ‘pilots’ have been established in several localities targeting high-risk populations^56,57^ with a commitment to expand the schemes under the NHS long-term plan.^58^ Concerns have been raised that the pilots, promoted as ‘lung health checks’, do not convey with sufficient clarity that participants are taking part in a screening programme^59^ and that their roll out has bypassed the UK National Screening Committee,^12^ which does not currently recommend population screening for lung cancer.^60^

**Table 1.** Summary of the National Lung Screening Trial and NELSON trial.

	NLST	NELSON^a^
Eligibility	Aged 55–74 years with 30 pack-year smoking history, who had not quit smoking within last 15 years	Male, aged 50–74 years who had smoked >15 cigarettes a day for >25 years or >10 cigarettes a day for >30 years, and had not quit >10 years ago
Number of randomised participants	53,454	13,195
Setting	United States	Belgium & Netherlands
Available study follow-up periods^b^	12.3 years (median)	10–11 years
Intervention and control	3 rounds of annual LDCT vs. 3 rounds of annual chest X-ray	4 rounds of LDCT (at 0, 1, 3 and 5.5 years) vs. no screening
Classification of test results^c^	Negative or positive Positive: any non-calcified nodule ≥4 mm	Negative, intermediate or positive Intermediate: nodules 50–500 mm^3^ Positive: nodules >500 mm^3^
Overall false-positive rate (% of positives)^c^	23.3% (96.4)	1.2% (56.5)
Positive predictive value	3.8% (95% CI: 3.4–4.3)	43.5% (95% CI: 38.9–48.1)
Relative risk reduction in lung cancer mortality	20.0% (95% CI: 8–27; p = 0.004)	24.0% (95% CI: 6–39; p = 0.01)
Number needed to screen to prevent 1 lung cancer death^d^	303 (at 12.3 years)	92–133 per round
Over-diagnosis rate^e^	3.1% (at 12.3 years)	8.9% (at 11 years)

^a^Results reported for NELSON pertain to male patients only, although a small sample of females were randomised and reported separate to the main results.

^b^Follow-up in original National Lung Screening Trial publication was for median 6.5 years, with a maximum duration of follow-up of 7.4 years in each group; however, subsequent analyses have been published based on additional follow-up.

^c^Abridged classification of results for NELSON which reflects first round of screening. For full nodule management protocol, see Xu et al.^15^

^d^Number needed to screen based on extended follow-up of 12.3 years. The original NLST trial publication reported number needed to screen of 320 based on initial medial follow-up of 6.5 years.

^e^The original National Lung Screening Trial publication reported over-diagnosis of 18.5% at 6 years. Nelson reported over-diagnosis of 19.7% at 10 years, reducing to 8.9% when follow-up was extended to 11 years.

## Is it cost-effective?

The National Lung Screening Trial was estimated to yield one quality-adjusted life year at a cost of $81,000^61^ while a more recent analysis estimated costs between $53,000 and $75,000 per quality-adjusted life year.^62^ While these estimates reflect the high cost of healthcare in the US, a systematic review found that a UK screening programme is unlikely to be cost-effective at a threshold of £20,000 per quality-adjusted life year.^63^ It is possible that an updated analysis, drawing on the findings of NELSON and extended follow-up data from the National Lung Screening Trial which showed fewer over-diagnosed cases, would produce lower cost estimates. Screening pilots have produced estimates of under £10,000 per quality-adjusted life year,^11,37^ possibly because of their focus on very high-risk populations. Due to significant staff shortages, particularly in radiology, the impact of diverting resources to screening activities on other diagnostic capacity also needs to be considered. Several strategies are being investigated to improve the cost-effectiveness of lung cancer screening including smoking cessation interventions embedded within screening programmes and efforts to determine the optimum interval between screens.

## No easy answers

The case for lung cancer screening is not straightforward. Lung cancer is a significant cause of morbidity, mortality and health inequality, with a disproportionate impact on deprived populations. Low-dose computed tomography screening in high-risk populations has been shown to significantly reduce lung cancer mortality.

Updating cost-effectiveness analyses with the findings of NELSON will help inform further policy decisions. Given that most people that develop lung cancer would either not be eligible for or would have chosen to attend screening, the likelihood that screening will benefit only a small proportion of participants, and the inherently controversial nature of investigating asymptomatic individuals, mean that decisions about implementing national screening programmes for lung cancer are unlikely to be settled with reference to cost-effectiveness alone.

Critics of screening have correctly asserted that it will always be much less cost-effective than smoking cessation interventions.^64^ We do not find this a coherent reason to dismiss lung cancer screening, since few interventions would reach that bar of cost-effectiveness. But we are also not persuaded that the case for lung cancer screening should be accepted as on the basis of equity with other cancers, due to similar performance to existing cancer screening programmes. Much more evidence has emerged about the harms and uncertainties of screening since the first programmes were introduced. The known harms of lung cancer screening are not trivial and include risks resulting from invasive procedures, the consequences of over-diagnosis and excess radiation.

It is precisely because such conundrums are so hard to unravel that the National Screening Committee was established to advice on UK screening policy. The National Screening Committee should have the opportunity to re-evaluate the evidence on lung cancer screening and make an assessment based on evidence from trials, cost-effectiveness and with reference to the values of potential participants. If deemed cost-effective and acceptable, a national screening programme could well form part of an effective strategy to reduce lung cancer deaths if implemented alongside adequately resourced measures to reduce smoking and continued emphasis on the detection and treatment of symptomatic cancers.

## Declarations

**Competing Interests:** The author(s) declared the following potential conflicts of interest with respect to the research, authorship, and/or publication of this article: Stephen H Bradley is undertaking a PhD on lung cancer diagnosis which is supported by Cancer Research UK. He is also a member of the executive committee of the Fabian Society which is a think tank affiliated to the Labour Party and is a member of the steering committee for a campaign to improve standards in health research (www.improvehealthresearch.com). Martyn PT Kennedy is a senior clinician and member of the trial management group for the Yorkshire Lung Cancer Screening Trial. Bethany Shinkins will be part-funded to provide statistical and methodology advice on a biomarker sub-study of the Yorkshire Lung Screening Trial, funded by Yorkshire Cancer Research.
